# ﻿*Ranunculusluanchuanensis* (Ranunculaceae), a new species from Henan, China

**DOI:** 10.3897/phytokeys.220.96799

**Published:** 2023-02-22

**Authors:** Wen-Qun Fei, Qiong Yuan, Qin-Er Yang

**Affiliations:** 1 Key Laboratory of Plant Resources Conservation and Sustainable Utilization, South China Botanical Garden, Chinese Academy of Sciences, Guangzhou 510655, Guangdong, China Key Laboratory of Plant Resources Conservation and Sustainable Utilization, South China Botanical Garden, Chinese Academy of Sciences Guangzhou China; 2 University of Chinese Academy of Sciences, Beijing 100049, China University of Chinese Academy of Sciences Beijing China; 3 Center of Conservation Biology, Core Botanical Gardens, South China Botanical Garden, Chinese Academy of Sciences, Guangzhou 510655, Guangdong, China Center of Conservation Biology, Core Botanical Gardens, South China Botanical Garden, Chinese Academy of Sciences Guangzhou China

**Keywords:** Asia, buttercups, Ranunculales, *Ranunculuslimprichtii*

## Abstract

*Ranunculusluanchuanensis* (Ranunculaceae), a new species from Laojun Shan in Luanchuan county, Henan province, central China, is here illustrated and described. It is morphologically similar to *R.limprichtii* in having 3-lobed and subreniform basal leaves, 3-lobed cauline leaves, and small flowers with reflexed and caducous sepals, but differs by having slender and basally slightly thickened roots (vs. fusiform), prostrate stems (vs. erect), obliquely ovoid and glabrous carpels and achenes (vs. widely ovoid and puberulous), longer styles in the carpels (ca. 1.2 mm vs. 0.6–0.8 mm) and achenes (ca. 1.8 mm vs. 0.6–0.8 mm), and glabrous receptacles (vs. sparsely puberulous). *Ranunculusluanchuanensis*, currently known only from its type locality, is geographically isolated from *R.limprichtii*, a species widely distributed in Gansu, Qinghai, Sichuan, Xizang (Tibet) and Yunnan, China. The distribution map of this new species and its putative closest ally, *R.limprichtii*, is also provided.

## ﻿Introduction

*Ranunculus* L., with ca. 600 species, is the largest genus in the Ranunculaceae and is widely distributed in all continents (Tamura 1995; Hörandl et al. 2005; Paun et al. 2005; Hörandl and Emadzade 2012). More than 150 species and 30 varieties of *Ranunculus* are currently recognized in China, one of the centers of species diversity for the genus (Wang 1995a, b, 1996, 2007, 2008, 2013, 2015, 2016, 2018, 2019a, b, 2022; Yang 2000; Wang and Gilbert 2001; Wang and Liao 2009; Luo and Zhao 2013; Wang and Chen 2015; Wang et al. 2016; Yuan and Yang 2017a, b, c; Zhang et al. 2020; Fei et al. 2022, 2023a, b). New species of *Ranunculus* have been frequently found and described due to intensive field investigations of once not easily accessible areas (Wang 2022; Fei et al. 2023a, b).

**Figure 1.**
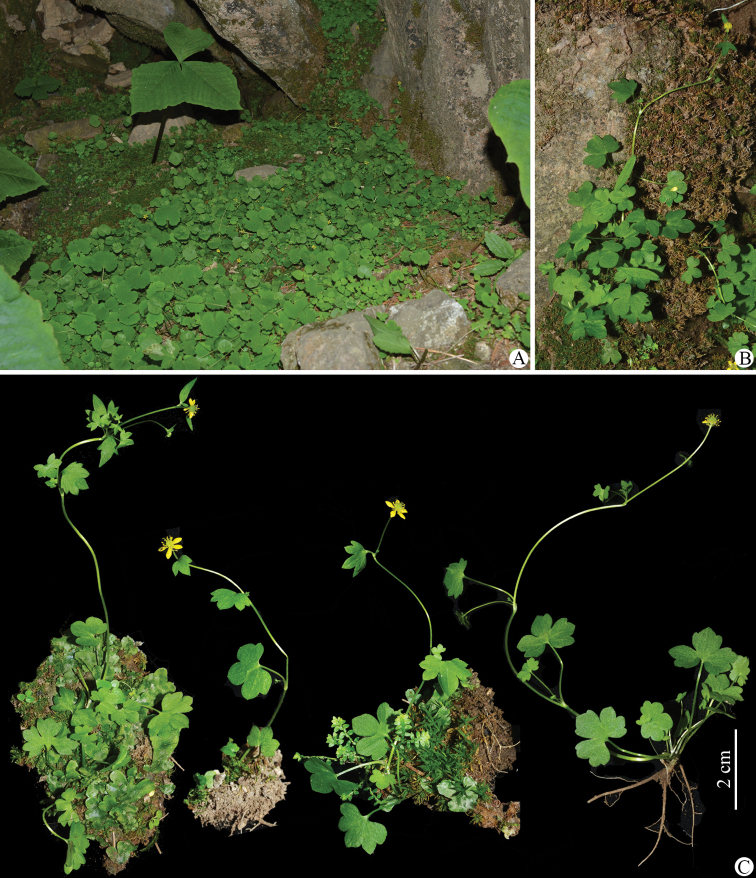
*Ranunculusluanchuanensis* sp. nov. in the wild (China, Henan, Luanchuan, Laojun Shan) **A, B** habitat **C** habit. Photographed by Wen-Qun Fei.

**Figure 2.**
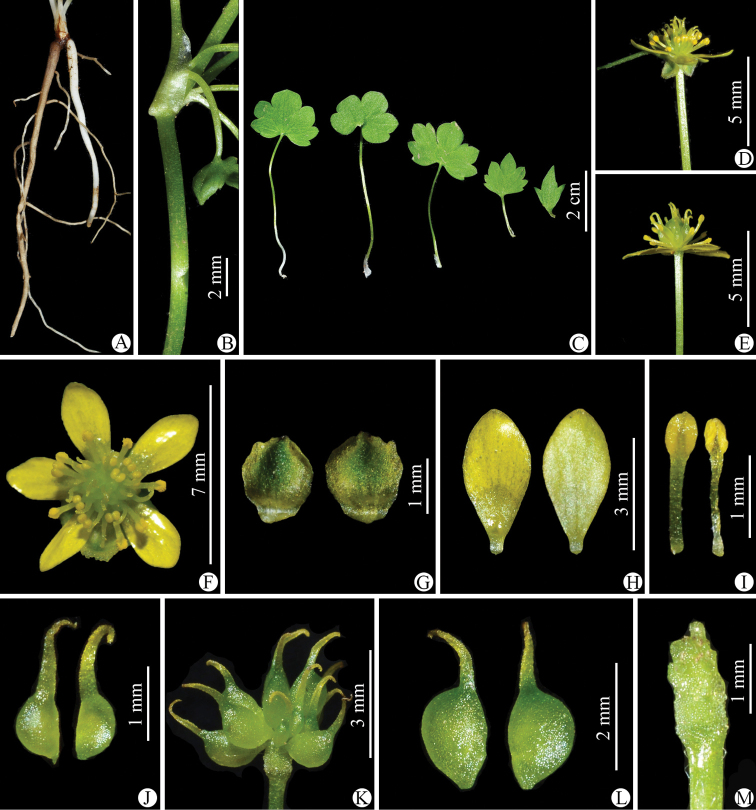
*Ranunculusluanchuanensis* sp. nov. in the wild (China, Henan, Luanchuan, Laojun Shan) **A** roots **B** portion of stem **C** leaves **D** flower with the sepals reflexed (lateral view) **E** flower with the sepals having fallen off (lateral view) **F** flower (top view) **G** sepal (left: abaxial side; right: adaxial side) **H** petal (left: adaxial side; right: abaxial side) **I** stamens **J** carpels **K** aggregate fruit **L** achenes **M** receptacle. Photographed by Wen-Qun Fei.

**Figure 3.**
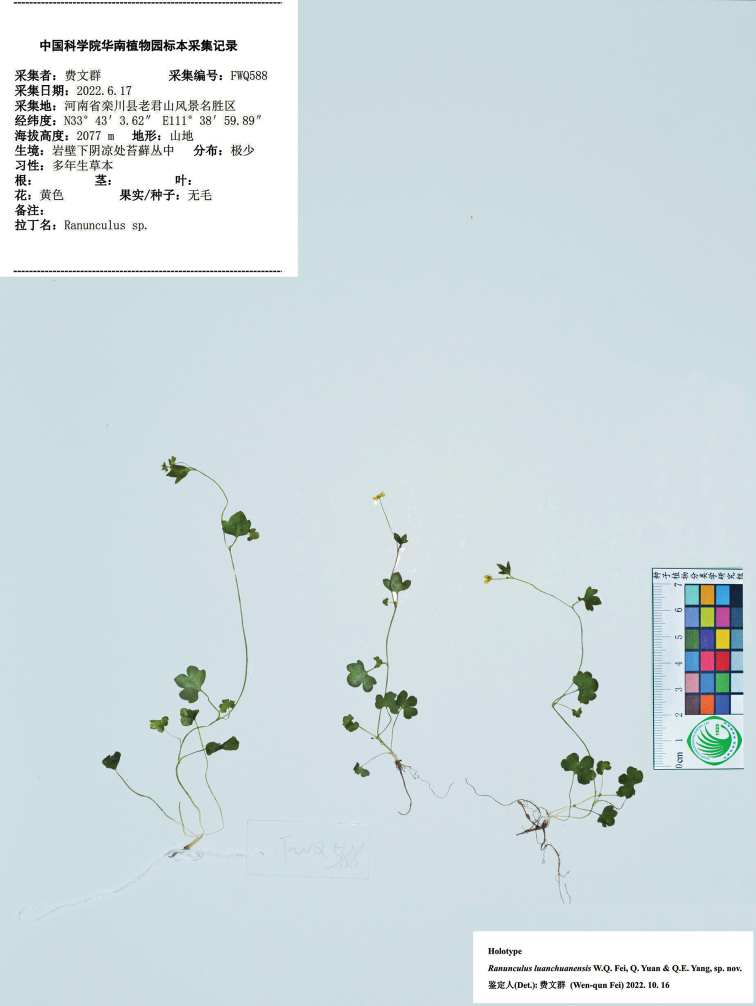
Holotype sheet of *Ranunculusluanchuanensis* sp. nov.

**Figure 4.**
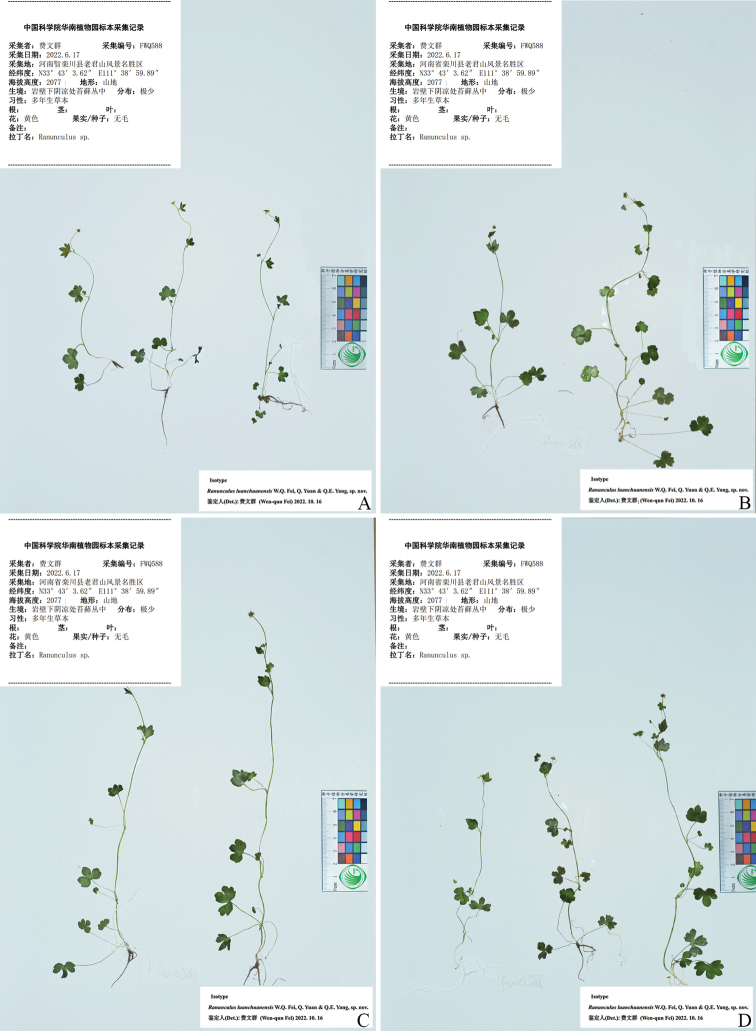
Isotype (**A–D**) sheets of *Ranunculusluanchuanensis* sp. nov.

**Figure 5.**
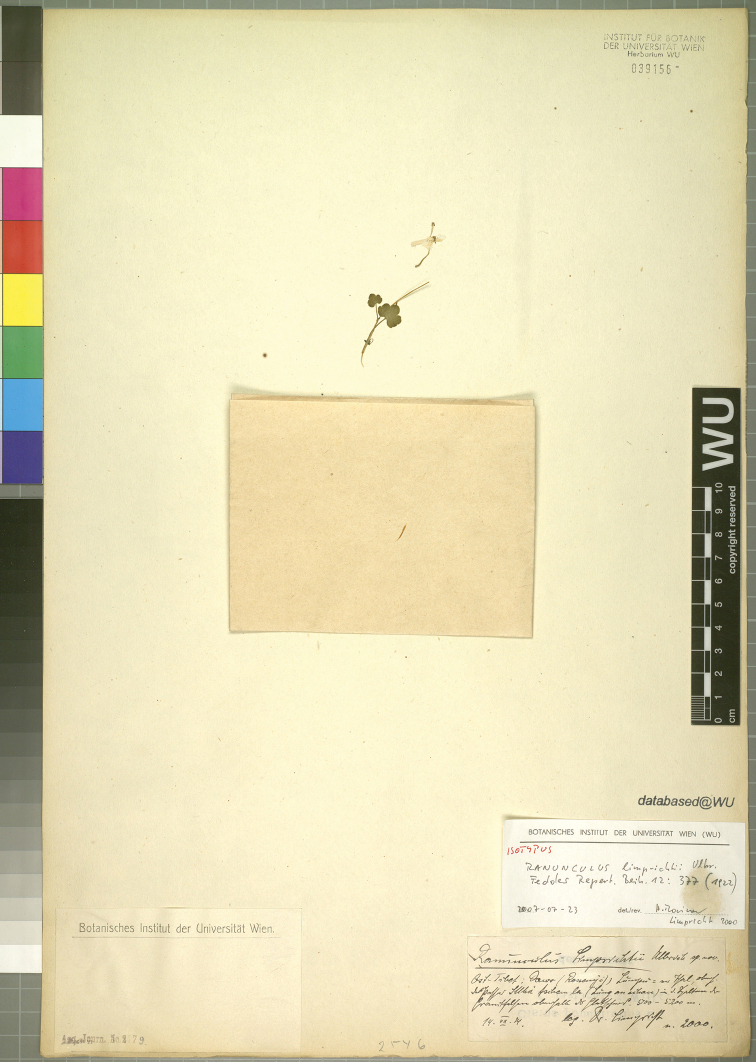
Isotype sheet of *Ranunculuslimprichtii*. Note that the holotype was most probably destroyed during World War II.

**Figure 6.**
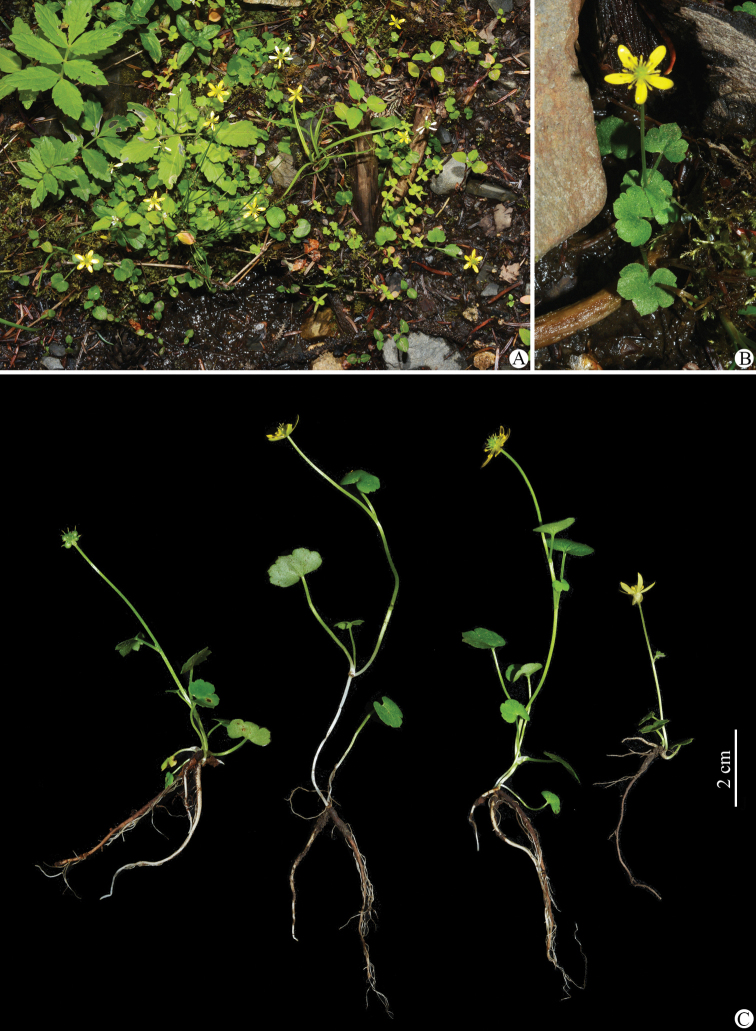
*Ranunculuslimprichtii* in the wild (China, Sichuan, Dawu, the type locality) **A, B** habitat **C** habit. Photographed by Wen-Qun Fei.

**Figure 7.**
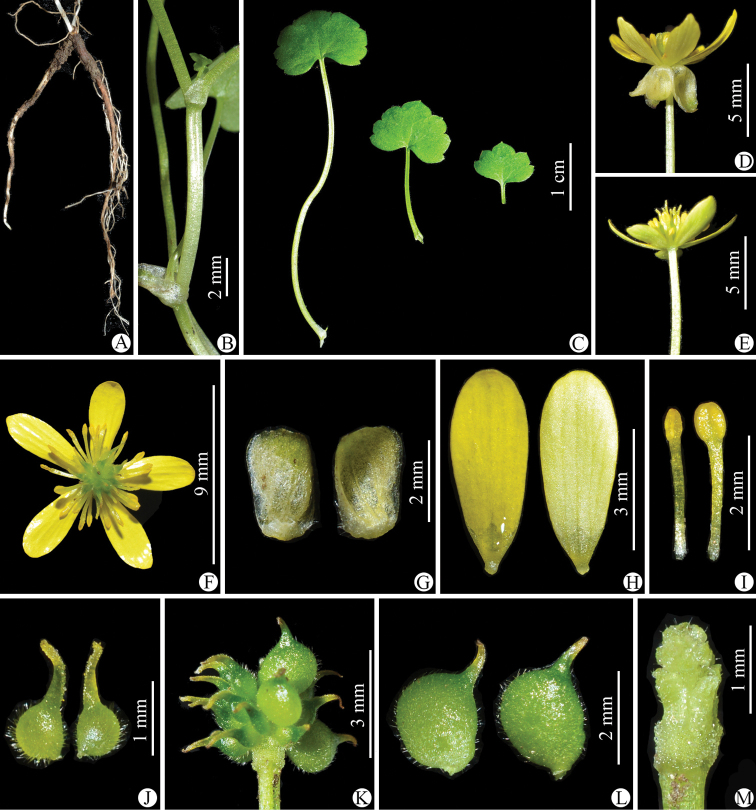
*Ranunculuslimprichtii* in the wild (China, Sichuan, Dawu, the type locality) **A** roots **B** portion of stem **C** leaves **D** flower with the sepals reflexed (lateral view) **E** flower with the sepals having fallen off (lateral view) **F** flower (top view) **G** sepal (left: abaxial side; right: adaxial side) **H** petal (left: adaxial side; right: abaxial side) **I** stamens **J** carpels **K** aggregate fruit **L** achenes **M** receptacle. Photographed by Wen-Qun Fei.

**Figure 8.**
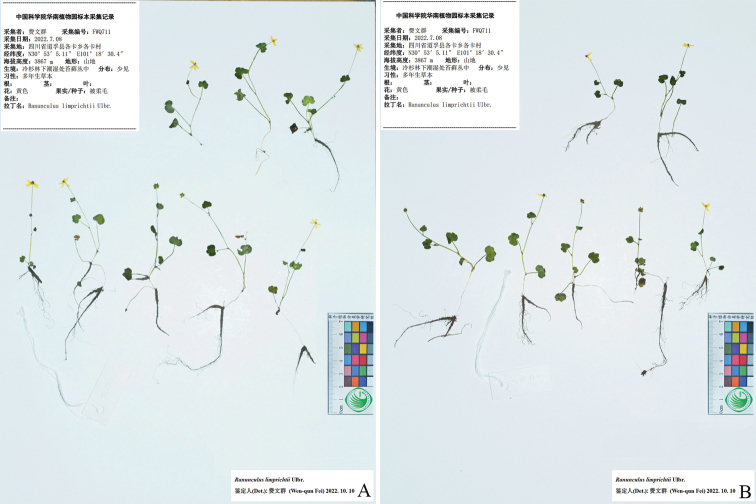
Selected specimens (**A, B**) of *Ranunculuslimprichtii* from Dawu in Sichuan province, China (the type locality).

**Figure 9.**
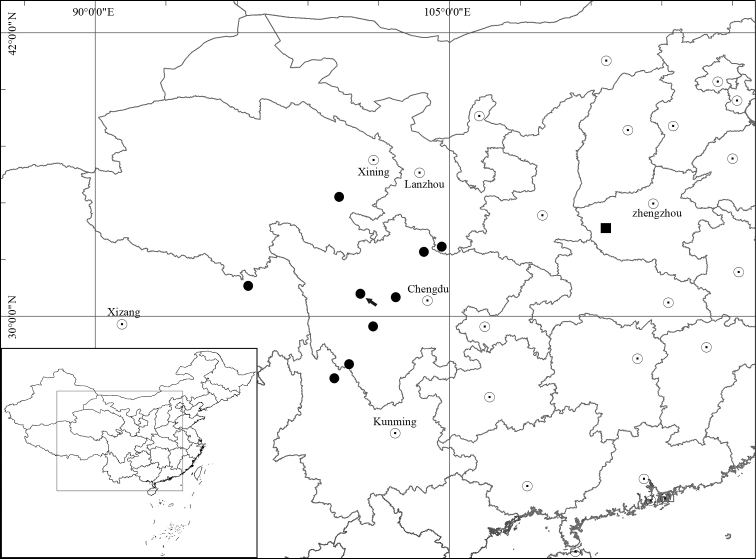
Distribution of *Ranunculuslimprichtii* (black circle) and *R.luanchuanensis* sp. nov. (black square). Black arrow indicates the type locality of *R.limprichtii*, i.e., Dawu in Sichuan province, China.

During our botanical expedition in June 2022 to Laojun Shan in Luanchuan county, Henan province, central China, we encountered an unusual population of *Ranunculus* (Figs 1–4). The plants grow in a shady area among boulders and have prostrate stems, 3-lobed and subreniform basal leaves, 3-lobed cauline leaves, small flowers with reflexed and caducous sepals, and glabrous carpels and achenes with long styles. They look like *R.limprichtii* Ulbr. (Figs 5–8) in having 3-lobed and subreniform basal leaves, 3-lobed cauline leaves (Figs 2C, 7C), small flowers (Figs 2D–F, 7D–F) with reflexed (Figs 2D, 7D) and caducous (Figs 2E, 7E) sepals, but differ by having slender and basally slightly thickened roots (vs. fusiform) (Figs 2A, 7A), prostrate stems (vs. erect) (Figs 1A, B, 6A, B), obliquely ovoid and glabrous carpels and achenes (vs. widely ovoid and puberulous) (Figs 2J, L, 7J, L), longer styles in the carpels (ca. 1.2 mm vs. 0.6–0.8 mm) (Figs 2J, 7J) and achenes (ca. 1.8 mm vs. 0.6–0.8 mm) (Figs 2L, 7L), and glabrous receptacles (vs. sparsely puberulous) (Figs 2M, 7M). A detailed morphological comparison between the two species is given in Table 1. *Ranunculuslimprichtii* is widely distributed in Gansu, Qinghai, Sichuan, Xizang (Tibet) and Yunnan, China (Fig. 9). Therefore, we determined that the population in question represents a hitherto undescribed species, which we name *R.luanchuanensis* and describe below.

**Table 1.** Morphological comparison between *Ranunculuslimprichtii* and *R.luanchuanensis* sp. nov.

	*R.limprichtii*	*R.luanchuanensis*
Roots	1‒3, fusiform	1‒3, slender, slightly thickened at base
Stems	single, 7‒10 cm tall, erect, glabrous or sparsely puberulous	single, 12‒20 cm long, prostrate, glabrous
Basal leaves	1(‒3)	2‒6
Flowers	terminal, 1, 8‒11 mm in diameter	terminal, 1(‒2), 6‒7 mm in diameter
Receptacles	ca. 1 mm long, clavate, sparsely puberulous	ca. 1 mm long, clavate, glabrous
Sepals	elliptic to obovate, abaxially sparsely puberulous	elliptic to obovate, abaxially sparsely puberulous
Petals	narrowly elliptic	narrowly obovate
Carpels	10‒15; ovaries widely ovoid, puberulous; styles 0.6‒0.8 mm long, apex recurved	14‒18; ovaries obliquely ovoid, glabrous; styles ca. 1.2 mm long, apex recurved
Aggregate fruit	subglobose	subglobose
Achenes	widely ovoid, puberulous, styles 0.6‒0.8 mm long	obliquely ovoid, glabrous, styles ca. 1.8 mm long

## ﻿Materials and methods

For morphological comparison, we examined physical specimens or high-resolution specimen images of *Ranunculuslimprichtii* at CDBI, HNWP, KUN, PE and WU (acronyms according to Thiers 2022). We also observed living plants in one population of *R.limprichtii* (Dawu in Sichuan province, the type locality) and one population of *R.luanchuanensis* (Luanchuan in Henan province). The morphological description of *R.luanchuanensis* was based on the observation of herbarium specimens and living plants in the wild.

## ﻿Taxonomy

### ﻿ Ranunculus luanchuanensis

Taxon classificationPlantaeRanunculalesRanunculaceae

W.Q.Fei, Q.Yuan & Q.E.Yang sp. nov.

14C622EE-F9C5-5471-A56E-9060322FD43A

urn:lsid:ipni.org:names:77314566-1

Figs 1 , 2 , 3 , 4

#### Diagnosis.

The new species is morphologically similar to *R.limprichtii* in having 3-lobed and subreniform basal leaves, 3-lobed cauline leaves, and small flowers with reflexed and caducous sepals, but differs by having slender and basally slightly thickened roots (vs. fusiform), prostrate stems (vs. erect), obliquely ovoid and glabrous carpels and achenes (vs. widely ovoid and puberulous), longer styles in the carpels (ca. 1.2 mm vs. 0.6–0.8 mm) and achenes (ca. 1.8 mm vs. 0.6–0.8 mm), and glabrous receptacles (vs. sparsely puberulous).

#### Type.

China. Henan province: Luanchuan county, Laojun Shan, 33°43'3.62"N, 111°38'59.89"E, in shady place among boulders on mountaintop, alt. 2077 m, 17 June 2022, *W.Q. Fei 588* (holotype: IBSC; isotypes: IBSC, PE).

#### Description.

***Herbs*** perennial, terrestrial or rupicolous. ***Roots*** 1–3, fibrous, slender, slightly thickened at base. ***Stems*** 12–20 cm long, prostrate, unbranched, glabrous. ***Basal leaves*** 2–6, 3-lobed, long petiolate; petioles 2–6 cm long, glabrous; blades 0.7–1.6 × 1–2.3 cm, subreniform in outline, thinly papery, adaxially green, glabrous or sparsely puberulous, abaxially light green, glabrous, base cordate, central segment 0.5–0.6 × 0.6–0.7 cm, widely obovate to rhombic-obovate, entire or 2–3-dentate, apex rounded or acuminate, lateral segments 0.5–0.7 × 0.7–1 cm, obliquely flabellate, inconspicuously 2-lobed, apex rounded or acuminate. ***Lower cauline leaves*** 2–3, similar to basal ones but smaller. ***Upper cauline leaves*** 1–2, 0.6–1.2 × 0.3–0.8 cm, 3-lobed, rarely entire, shortly petiolate or subsessile, oblate-ovate, flabellate or lanceolate, glabrous. ***Inflorescences*** terminal, 1(–2)-flowered. ***Flowers*** 6–7 mm in diameter; pedicels 0.5–2 cm long, glabrous or sparsely puberulous; receptacles ca. 1 mm long, clavate, glabrous; sepals 5, 2–2.5 × 1.5–1.8 mm, elliptic to obovate, reflexed, caducous, green tinged with yellowish, concave, adaxially glabrous, abaxially sparsely puberulous; petals 5(–6), 3–3.5 × 1.5–1.8 mm, narrowly obovate, yellow, glabrous, apex obtuse or acuminate, nectary pit without a scale, claws ca. 0.4 mm long; stamens 12–16, filaments ca. 1.5 mm long, narrowly linear, anthers ca. 0.5 mm long, oblong; gynoecium subglobose; carpels 14–18, ovaries ca. 0.8 mm long, obliquely ovoid, laterally flattened, biconvex, glabrous, styles ca. 1.2 mm long, glabrous, apex recurved. ***Aggregate fruit*** ca. 3.5 × 3.5 mm, subglobose; achenes ca. 1.8 × 1.2 mm, obliquely ovoid, laterally flattened, biconvex, glabrous, styles ca. 1.8 mm long, persistent, apex recurved.

#### Etymology.

The specific epithet refers to the type locality of the new species, i.e., Luanchuan county in Henan province, central China.

#### Phenology.

Flowering in early June; fruiting at the end of June.

#### Distribution and habitat.

*Ranunculusluanchuanensis* is currently known only from its type locality, i.e., Laojun Shan in Luanchuan county, Henan province, central China (Fig. 9). It grows in a shady area among boulders on a mountaintop at an altitude of 2077 m above sea level.

#### Conservation status.

*Ranunculusluanchuanensis* is currently known only from one small population at its type locality, i.e., Laojun Shan in Luanchuan county, Henan province, central China. This population consists of ca. 100 individuals within an area of less than 3 m^2^. However, the threat risk seems low because this species is not economically valuable and grows in a secluded place. The conservation status of *R.luanchuanensis* is here categorized as “Data Deficient (DD)” before adequate information on this species is acquired (IUCN Standards and Petitions Committee 2022).

#### Notes.

Since its description, Ranunculuslimprichtiivar.flavus Hand.-Mazz. has been known only from its type material from Songpan county in Sichuan province, China (Wang 1995b). Based on our observations of herbarium specimens and living plants in the wild, we agree with Liou (1980) that this variety should be reduced to the synonymy of *R.limprichtii*. We will deal with the identity of R.limprichtiivar.flavus in detail elsewhere.

According to Tamura’s (1995) infrageneric classification of *Ranunculus*, *R.luanchuanensis* should be assigned to R.sect.Ranunculus, which is characterized by having swollen achenes with a distinct beak and receptacles hardly enlarged after anthesis. *Ranunculuslimprichtii*, the putative closest ally of *R.luanchuanensis*, was placed by Wang (1995b) in R.sect.Ranunculus, with the section being incorrectly treated by him as R.sect.Auricomus (Spach) Schur. We accept the sectional placement of *R.limprichtii* since it is in accordance with the current placement of our new species in the same section.

## Supplementary Material

XML Treatment for Ranunculus luanchuanensis
